# The relationship of maternal anxiety, positive and negative affect schedule, and fatigue with neonatal psychological health upon childbirth

**DOI:** 10.1186/s40834-021-00155-8

**Published:** 2021-04-01

**Authors:** Sara Dokuhaki, Fateme Dokuhaki, Marzieh Akbarzadeh

**Affiliations:** 1 Community Based Psychiatric Care Research Center, Department of Midwifery, School of Nursing and Midwifery, Shiraz University of Medical Sciences, Shiraz, Iran; 2 Department of Psychology, Islamic Azad University of Marvdasht, Marvdasht, Iran; 3 Maternal-fetal Medicine Research Center, Department of Midwifery, School of Nursing and Midwifery, Shiraz University of Medical Sciences, Shiraz, Iran

**Keywords:** Anxiety, Mood, Fatigue, Infant, Health

## Abstract

**Background:**

Exposure of mothers to negative moods and stress before childbirth leads to negative consequences for the infants. Given the importance of psychological health, this study aimed to examine the effect of these factors on the infants’ psychological health.

**Method:**

This cross-sectional study was conducted in Shiraz hospitals on 110 pregnant women selected with multistage random sampling. Research tools included The McGill Pain Questionnaire (MPQ) to measure fatigue with three criteria; The Positive and Negative Emotion Schedule (PANAS); and The Spielberger State-Trait Anxiety Inventory (STAI) were used to measure maternal mood and anxiety level. Also, neonatal psychological health was assessed by a checklist. Neonatal psychological health’s correlation with maternal anxiety, fatigue, and mental state was assessed. Data were analyzed by SPSS-19 software using Pearson correlation coefficient and statistical regression at the significance level of 0.05.

**Result:**

Although there was no significant relationship between maternal anxiety score and neonatal psychological health after birth (*p* = 0.231; *r*=-0.343), the relationship was significant immediately after birth with positive (*P* < 0.001; *r* = 0.343) and negative affect scores (*P* < 0.001; *r*=-0.357).

**Conclusions:**

There was a statistically significant relationship between the neonatal psychological health and maternal fatigue (*p* ≤ 0.001; *r* = -0.357) and PANAS (*p* ≤ 0.001) of the mother; however, it had no significant relationship with maternal anxiety (*p* = 0.231; *r*=- 0.343). Therefore, nurses and midwives can reduce maternal anxiety and improve neonatal mental health by supporting mothers.

## Introduction

Anxiety, depression, stress and excitement of pregnancy period, especially upon childbirth, are usually overlooked in women [1]. Anxiety and stress have deleterious effects on the mother and infant [2]. Studies show that exposure of the mother to negative excitement and stress before childbirth increases the risk of behavioral and psychological problems in the infant’s life after birth. Maternal psychological stress is basically known as a teratogen, a factor that can entail deleterious prenatal consequences [3, 4]. Higher pregnant mothers’ anxiety scores are associated with the higher likelihood of cesarean childbirth and lower successful breastfeeding [5]. Anxiety gives rise to lower Apgar scores of infants upon birth and at minute 5; in this regard, Stuart et al. reported that low Apgar scores at minute 5 after birth were associated with a slight cognitive disorder in didactic performance [6].

In fact, the human embryo is extremely sensitive to maternal stress and affective moods. Post-natal stress can affect birth results and may cause neurobehavioral development in damaged infants. In a study, training on decreasing anxiety and increasing fetal affection toward pregnant mothers during the pregnancy increased neonatal psychological health upon birth [7, 8].

Neonatal exposure to maternal stress before birth was associated with low birth weight and smaller head perimeter. Improved maternal mood and calmness, and thus more neonatal improvement results in quicker attainment of motor development indicators [9, 10]. Similarly, physical indicators (weight, height, and head perimeter) were significantly greater than those of infants whose mothers have not received the necessary training during pregnancy [11, 12], which is due reduced anxiety, improved mood, and more kindness toward the fetus.

Several studies also showed that maternal stress reduced neonatal cognitive functions and brain volume, leading to learning and memory problems. Similarly, some reports on maternal anxiety or childbirth anxiety pointed out the emotional problems, hyperactivity, attention deficiency, and Tourette syndrome among such children.

Pregnancy stress appears to be one of the determinant factors of delayed growth and mental progress in 8-month infants and might be a risk factor for subsequent developmental problems.

On the other hand, in addition to anxiety and stress, mothers experience fatigue during pregnancy and after birth [13, 14]. In fact, during pregnancy, fatigue mostly comes with depression, anxiety, fear, and long duration of work; therefore, it can bring about similar effects, i.e. stress-derived fatigue and pregnant women usually report fatigue (87.2 to 96.5) [15]. Also, prenatal fatigue comes with postnatal depression, anxiety, premature childbirth, and cesarean section. In other words, maternal fatigue and anxiety have similar effects on the women’s and consequently, infants’ health; and psychological health problems during childhood will be a major challenge for their general health in the future [16].

That is often the main reason for impaired learning and social disorder that can also continue with greater severity at older ages; as the studies of neural sciences, biology, genetics, and social sciences within the past two decades have shown that the first 1000 days of life (childbirth period and first two years of life) is the golden age of brain development and psychological health of the infant [17–19]. Most of the studies revealed the relationship between deleterious effects of stress, fatigue, and mother’s mood on neonatal physical health, growth, and development. The detrimental effects on neonatal psychological health indicators have not been addressed sufficiently [20–23], which are dealt with in the present study.

## Materials and methods

This descriptive study (correlation type) was conducted on 110 pregnant women aged 18–40 years old, who have referred to the hospital for labor. The inclusion criteria were gravida one, singleton and term pregnancy, and natural childbirth. Thus, mothers lacking severe and chronic diseases and willing to take part in the study were enrolled. The exclusion criteria were suffering from any physical or mental problems. The mothers were selected from the maternity ward of Hazrat Zeinab (P.B.U.H), Shoushtari, and Hafez hospitals affiliated with Shiraz University of Medical Sciences using random multistage sampling. The degree of anxiety was measured using STAI. Also, positive and negative emotion scores and pregnant mothers’ fatigue were measured through standard questions. Neonatal psychological health was determined after birth using questionnaires related to the neonatal psychological health upon birth. Data were analyzed with SPSS software using the Pearson correlation coefficient and statistical regression at the significance level of 0.05.

The research tools used in this study were as follows:

1. Spielberger State-Trait Anxiety Inventory (STAI): It is used for assessing maternal anxiety level. It has 40 items, 20 of which are about positional anxiety and 20 are about character anxiety, and are scored based on a 3-point Likert scale. A total score of 0–19 indicates no anxiety; 20–40 indicates slight anxiety; 41–60 indicates average anxiety; and 61–80 indicates severe anxiety. Aqamohammadi et al. (2007) used STAI on 150 patients under surgery and reported a reliability of 97 %. The validity and reliability of the questionnaire have been reported by Aqamohammadi et al., which was the basis of the present study [24].
2. The McGill Pain Questionnaire (MPQ): It is used to measure measuring the affective status and fatigue with three mental, cognitive, psychological criteria .
3. The Positive And Negative Affect Schedule (PANAS) is used for assessing the participants’ mood at a specific time (like the time of conducting the test). It is made up of 20 words, each of which denotes different feelings and emotions, which are scored based on a 3-point Likert scale from very low or not at all to very high. The positive and negative PANAS are obtained from the sum of the scores of ten positive and ten negative words, respectively. Positive and negative words are retrieved in a scattered manner. In this study, the Cronbach alpha and test-retest correlation for the positive section of PANAS were 0.88 and 0.85, respectively. These were 0.87 and 0.89 for the negative part of PANAS, respectively.
4. Neonatal psychological health assessment checklist upon childbirth is developed based on the neonatal psychological health indicators derived from psychological health and development of psychology and pregnancy books. Upon childbirth, the neonatal psychological health indicators cannot be separated from its physical health, so an attempt was made to measure the main indicators of psychological health using these questions.

The checklist upon childbirth has 9 questions, scores 0, 1 or 2.; Finally, the scores of the 9 questions are summed up; the overall score of the checklists ranges from 0 to 18. Higher scores indicate higher neonatal psychological health. Cronbach alpha and split-half scores for this checklist were 0.89 and 0.72, respectively. The content validity of the checklist was calculated through scoring and assessing by 6 pediatricians. Given the obtained validity and reliability derived from the study conducted by Shayeqhian et al., we used this questionnaire in this study [25].

## Results

The mean age of the participants in this study was 26.42 ± 5.60 years; their mean anxiety level was 29.99 ± 14.05, their mean fatigue was 38.37 ± 16.11, their positive emotion was 37.22 ± 7.33, and mean neonatal psychological health upon birth was 16.68 ± 1.35 (Table 1). The Kolmogorov Smirnov test was used to assess data normality which indicated normal distribution of all data. Therefore, the Pearson correlation coefficient was used for obtaining the linear relationship, which showed no statistical relationship between maternal anxiety score and neonatal psychological health after birth. Moreover, there was a statistically significant relationship between neonatal psychological health score immediately after birth and a positive emotion score (*P* < 0.001; *r* = 0.516) and maternal fatigue score (*p* < 0.001; *r*=-0.357) (Table 2).

**Table 1:** Neonatal Mental Health Assessment by Maternal Anxiety Level

Maternal Anxiety Score	N	Mean ± SD Neonatal Mental Health	95 % Confidence Interval for Mean		Minimum	Maximum
			**Lower bound**	**Upper bound**		
**Normal**	33	16.87 ± 1.24	16.43	17.31	14	18
**Mild**	50	16.68 ± 1.46	16.26	17.09	13	18
**Medium**	27	16.44 ± 1.28	15.93	16.95	14	18
**Severe**	0	-	-	-	-	-
**Total**	110	16.68 ± 1.35	16.42	16.93	13	18

**Table 2:** Pearson Correlation with Neonatal Mental Health at Birth

Items	Mean ± SD	Min	Max	Pearson correlation coefficient with mental health	*P* value
**Anxiety score**	29.99 ± 14.05	6	60	ˍ 0.115	0.231
**Positive PANAS score**	37.22 ± 7.87	15	50	0.343	< 0.001
**Negative PANAS score**	18.55 ± 7.33	10	38	ˍ 0.574	< 0.001
**Total PANAS score**	18.67 ± 13.38	­ˍ 16	40	0.516	< 0.001
**Fatigue**	38.37 ± 16.11	0.00	71.67	ˍ 0.357	< 0.001
**Neonatal Mental Health**	16.68 ± 1.35	13	18	1	-

## Discussion

The present study found no significant relationship between maternal anxiety and neonatal psychological health after birth (*r*=-0.115; *p* = 0.231), or neonatal psychological health scores including Apgar score, and birth weight. Keenan et al. also reported that birth weight and Apgar score were not related to maternal anxiety and depression [26].

However, Hassanjanzadeh et al. emphasized the relationship of neonatal outcomes with pregnancy stress and depression and anxiety during pregnancy [27]. In their study, the results of correlation of depression, stress, and anxiety variables with birth weight, length and perimeter of the head, and Apgar score of infants showed significant differences (*P* < 0.05). Multiple regression analysis showed that interpersonal relationship was correlated with birth weight prediction (B=-0.324), anxiety with height prediction (B=-0.197); stress with head perimeter prediction (B=-0.350); and depression with Apgar score prediction (B=-0.323; *p* < 0.001).

Although this study found an obvious relationship between neonatal psychological health and other emotional maternal indicators such as maternal mood and fatigue upon childbirth, measurement should be carried out on a periodical basis and during pregnancy, not only upon childbirth since maternal anxiety diagram within pregnancy is U-shape, while in this study the anxiety experienced by the mothers during the early postpartum months was not taken into account. Similarly, in the study carried out by Zijlmans et al., it was indicated that pregnancy anxiety and increased cortisol levels in pregnant mothers were associated with respiratory diseases and children’s digestion problems until the age of 3 [28]. These findings showed that the possible effects of anxiety and negative mood of mothers extend beyond the first year of life and into the childhood.

Fan et al. reported a direct relationship between maternal mood and neonatal health and indicated that maternal anxiety had deleterious effects on the infants including their birth weight. Their results showed that the long-term effect of maternal mood during pregnancy leads to cardiovascular responses among infants [29]. In a study carried out by Chien et al., increased fatigue elevated the rate of cesarean Sec. [14]. Also, in our study, the extent of mothers’ fatigue was considered as one of the factors affecting neonatal psychological health.

Staneva et al. in their study entitled “the effect of mothers’ depression, anxiety and perceived stress during the pregnancy period on the premature accouchement” concluded that depression, anxiety, and stress of pregnant women had a significant relationship with detrimental consequents and premature accouchement in infants [30]. Distress and undesired mood effects within the mother’s pregnancy are routinely characterized with premature childbirth but without medical etiology. Overall, we could find no similar studies investigating the relationship of the neonatal psychological status with reduction of maternal fatigue and anxiety, and improvement of mood during the first 24 h after vaginal childbirth. However, several studies have been conducted on post-cesarean and vaginal delivery pain, fatigue, and maternal mood [30–34]. The present study had some limitations. Participants were selected from only two childbirth centers. This group of participants may not be representative of the target population. The sample size was small, and we did not control some confounding variables such as delivery time and the psychological status during pregnancy. Therefore, studies with larger sample sizes and longer duration are recommended.

## Conclusions

There was a significant relationship between the neonatal psychological health and the scores of mothers’ PANAS mood and fatigue; however, no significant relationship was found with maternal anxiety. Therefore, healthcare personnel like physicians, midwives, nurses, and psychologists who work in the field of pregnancy caregiving should support pregnant mothers during accouchement and childbirth. Also, supporting mothers can result in improvement of maternal outcomes and neonatal psychological health.

## Data Availability

Data are available upon request from the corresponding author.
